# Comparative evaluation of insertion torque and mechanical stability for self-tapping and self-drilling orthodontic miniscrews – an in vitro study

**DOI:** 10.1186/s13005-017-0143-3

**Published:** 2017-05-30

**Authors:** Michele Tepedino, Francesco Masedu, Claudio Chimenti

**Affiliations:** Department of Biotechnological and Applied Clinical Sciences, University of L’Aquila, Viale S.Salvatore, Edificio Delta 6, 67100 L’Aquila, Italy

**Keywords:** Miniscrew, Implant design, Orthodontic mini-implant, Insertion torque, Stability

## Abstract

**Background:**

The aim of the present study was to evaluate the relationship between insertion torque and stability of miniscrews in terms of resistance against dislocation, then comparing a self-tapping screw with a self-drilling one.

**Methods:**

Insertion torque was measured during placement of 30 self-drilling and 31 self-tapping stainless steel miniscrews (Leone SpA, Sesto Fiorentino, Italy) in synthetic bone blocks. Then, an increasing pulling force was applied at an angle of 90° and 45°, and the displacement of the miniscrews was recorded.

**Results:**

The statistical analysis showed a statistically significant difference between the mean Maximum Insertion Torque (MIT) observed in the two groups and showed that force angulation and MIT have a statistically significant effect on miniscrews stability. For both the miniscrews, an angle of 90° between miniscrew and loading force is preferable in terms of stability.

**Conclusions:**

The tested self-drilling orthodontic miniscrews showed higher MIT and greater resistance against dislocation than the self-tapping ones.

**Electronic supplementary material:**

The online version of this article (doi:10.1186/s13005-017-0143-3) contains supplementary material, which is available to authorized users.

## Background

Anchorage management is often an issue in orthodontic treatment, and many devices and solutions are offered to deal with different clinical situations. In some cases an “absolute anchorage” is needed to dissipate all unwanted reaction forces: such a task can be afforded by orthodontic miniscrews.

Orthodontic miniscrews (also known as microscrews, micro/mini-implants, orthodontic implants or TADs —temporary anchorage devices) are devices specially designed to be placed within the maxillofacial bones with the aim of providing anchorage for an orthodontic appliance [1]. They can have different diameters and lengths, body designs and thread shapes, and can be made of different alloys (basically grade 5 titanium alloy or stainless steel). Depending on the insertion technique, orthodontic miniscrews can be divided into self-drilling screws (which have a cutting tip that determines a drill-like action during placement) and self-tapping ones (which have a non-cutting tip and require pre-drilling of the surgical site to create a pilot hole).

Orthodontic miniscrews can be helpful in many situations: when patient compliance is an issue, when teeth are insufficient to assure appropriate biomechanics, or when anchorage management is critical [1].

Orthodontic miniscrews are designed for temporary usage, so in most cases osseointegration of the screw is unwanted in order to facilitate its removal, and only a primary stability is pursued [2]. Such devices are widely used in orthodontic practice and their success rate is reported to be between 37% and 94% [3–5]: many factors affect success rate, like miniscrew design, intra-oral position, surgical technique and loading. According to Melsen and Costa [6], primary stability is one of the key factors for clinical success. Stability is a variable that can be evaluated with quantitative methods, such as periotest, resonance frequency analysis, pullout test or insertion/removal torque recording [7]. During the placement of miniscrews, it is necessary to have a certain amount of insertion torque to achieve good primary stability [7, 8]: however, an excessive torque can lead to fractures in the cortical bone and bone resorption, hence to failure of the miniscrew [9]. Values of Maximum Insertion Torque (MIT, the maximum value of insertion torque registered during the insertion of the miniscrew) ranging from 5 to 10 N·cm have been presented as a reference in many articles [7–10]. A systematic review of the literature has been conducted to assess the correlation between insertion torque and success of miniscrews [7]: only non-randomized studies were judged eligible for the revision, and no evidence was found indicating the ideal insertion torque for achieving clinical success. Moreover the authors concluded that further high quality studies are required, and that there is the need to analyse individually each factor that can affect maximum insertion torque, starting from laboratory models.

The aim of the present work was to evaluate the relationship between insertion torque and stability of miniscrews in terms of resistance against a dislocating force applied at different angulations, then comparing the behaviour of a self-tapping miniscrew with a self-drilling one.

## Methods

**Fig. 1:**
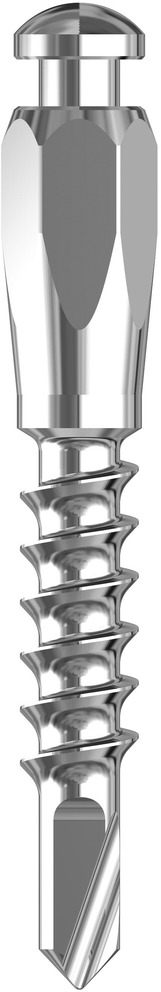
Self-drilling mini-implant used in the study

**Fig. 2:**
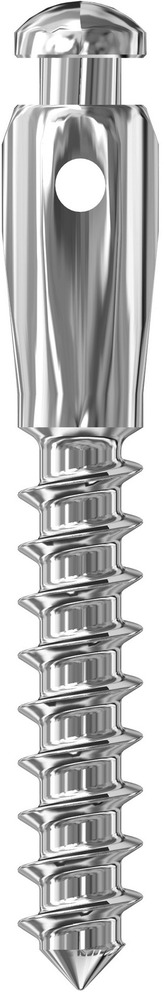
Self-tapping mini-implant used in the study

Sixty-one stainless steel orthodontic miniscrews (Leone SpA, Sesto Fiorentino, Italy) were used, 30 self-drilling (Fig. 1) and 31 self-tapping (Fig. 2). Both types of miniscrew had inner diameter of 1.3 mm, pitch of 0.8 mm, cylindrical trunk shape, length of 8 mm and high head (worse biomechanical condition). Self-tapping mini-implants had an outer diameter of 2 mm, while self-drilling ones had an outer diameter of 1.75 mm.

Synthetic bony blocks made of rigid polyurethane foam (Sawbones, Pacific Research Laboratories Inc., Vashon, WA, USA) were used. Each bone block was composed of a cortical layer with a density of 0.64 g/cm^3^ (40 pcf) and a thickness of 2 mm, and a cancellous bone layer with a density of 0.32 g/cm^3^ (20 pcf). Such a configuration was chosen to simulate the bone quality of a maxillary premolar region, as reported by other authors [2].

**Fig. 3:**
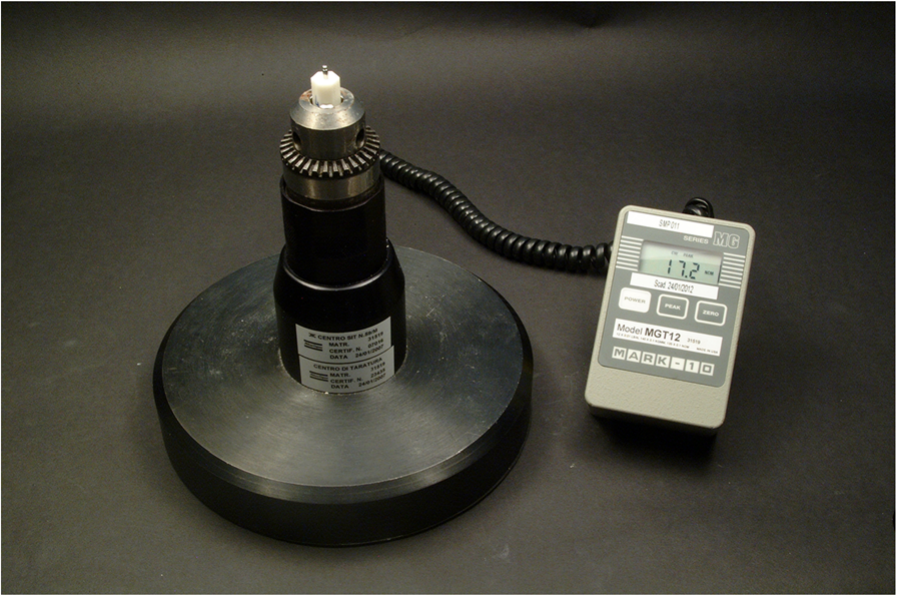
Digital torque gauge

The synthetic bone blocks were placed over a digital torque gauge (MGT12E, Mark-10, New York, USA) (Fig. 3) and the miniscrews were positioned, in order to measure the maximum insertion torque (MIT). Care was taken to ensure that the insertion site of the screw was aligned with the centre of the platform. The torque gauge was calibrated before each measurement.

Both types of miniscrew were positioned in a block with a 30:1 contra-angle mounted over a surgical engine (ChiroPro 980, Bien-Air Dental, Bienne, Switzerland) at a rotating speed of 15 rpm, taking care to place the screw tilted at an angle of 90° with respect to the surface of the block and fully inserting the miniscrew till the end of the threaded portion. The insertion of the self-tapping screws, however, required site preparation using a 1.5-mm-diameter pilot drill with a rotational speed of 300 rpm.

**Fig. 4:**
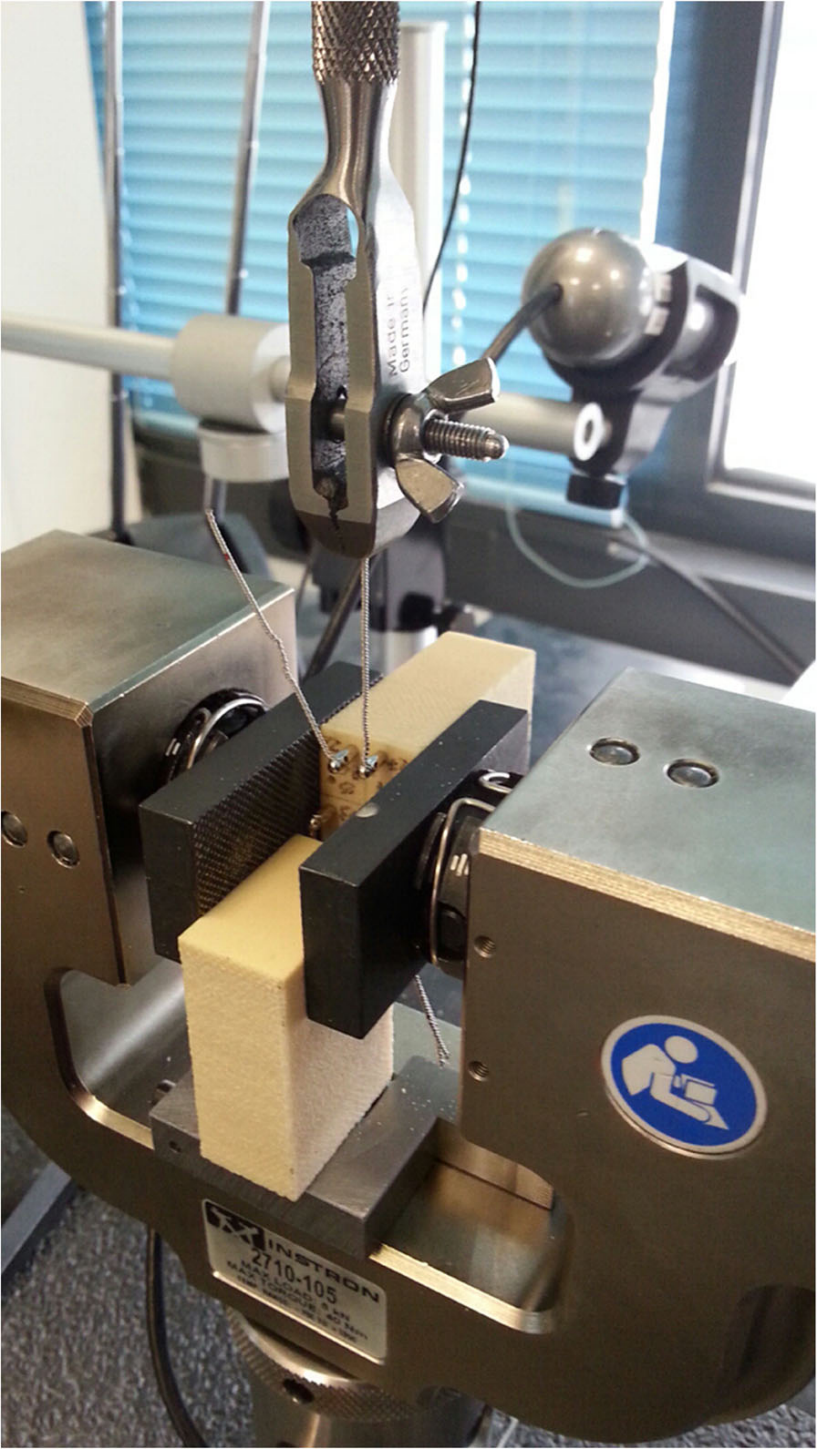
Image showing how the bone blocks and the miniscrews were positioned into the Instron machine to test displacement under loading force

**Fig. 5:**
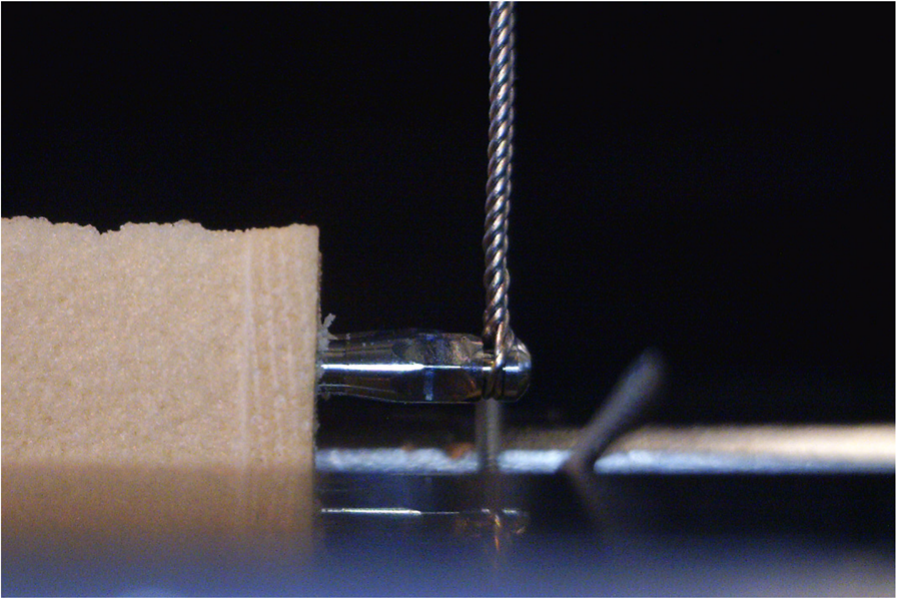
Miniscrew placed in a synthetic bone block and positioned into the Instron machine in the 90° configuration

In order to apply the displacing force and then measure the miniscrew displacement, an Instron® mechanical testing machine (3365 Series, Instron®, Norwood, USA) with a loading cell of 100 N was used (Fig. 4). The specimens were secured to the testing machine and connected to the loading cell through a 0.12 in. stainless steel orthodontic ligature (Leone SpA, Sesto Fiorentino, Italy) by the same operator (MT) in such a way that force was applied either at an angle of 90° (Fig. 5) or 45° in the opposite direction of the pulling force. All the specimens were tested in both configurations.

The loading cell was set to run for a maximum distance of 1 mm, with an increasing force. When the applied force reached the value of 20 N, the distance covered by the loading cell was measured and stored: such a distance corresponds to the sum of the mini-implant’s displacement and of the wire ligature’s elongation. The loading cell was calibrated before each measurement.

Preliminary tests had been performed to evaluate the elongation of the stainless steel wire used for the testing of dislocation and to test the reproducibility of the operator who made the wire ligatures: the elastic deformation of the orthodontic wire was subtracted from the total loading cell displacement, thus obtaining the pure displacement of the miniscrews.

### Statistical analysis

A statistical analysis of the data was performed. Descriptive statistics, mean values and standard deviations of the variables considered were computed. First type error was set at 0.05.

A preliminary Hotelling test was carried out to assess any overall difference between MIT and the screw displacement with respect to miniscrew type.

The effect of the miniscrew type on the variable MIT was assessed and estimated by a linear regression model, and likewise a linear regression model was used to address the behaviour of the screw displacement with respect to force angulation and insertion torque.

Model fitting was estimated by F test. The analysis was carried out by STATA software version 13 (StataCorp LP, Texas, USA).

## Results

Descriptive statistics are reported (Tables 1 and 2). Mean values for MIT were higher for self-drilling screws (6.43 ± 2.09 N·cm), while self-tapping ones showed higher mean values for each screw displacement (0.37 ± 0.08 mm). Miniscrews positioned at an angle of 45° in the opposite direction of applied force had higher mean values of displacement (0.38 ± 0.06 mm) than those placed at 90°.

**Table 1:** Mean values and standard deviations (SD) for maximum insertion torque (MIT) divided by miniscrew’s type

	Self-drilling		Self-tapping	
	Mean	SD	Mean	SD
MIT (N·cm)	6.43	2.09	4.12	1.21

**Table 2:** Mean values and standard deviations (SD) for miniscrew displacement at a force of 20 N, divided by miniscrew’s type and angulation of force applied

	Self-drilling				Self-tapping			
	90°		45°		90°		45°	
	Mean	SD	Mean	SD	Mean	SD	Mean	SD
Displacement (mm)	0.30	0.04	0.36	0.04	0.35	0.09	0.40	0.08

The statistical analysis showed a statistically significant difference between the mean MIT observed in the groups with different screws, yielding an average difference between self-drilling and self-tapping screws of 2.31 ± 0.43 N·cm (*p* < 0.05) (Table 3).

**Table 3:** Linear regression model for maximum insertion torque (MIT) by miniscrew’s type

MIT vs Miniscrew type Linear Model*						
Covariates	$\widehat{\beta}$_i_	$\widehat{\sigma}$_i_	t	*p*_i_	Conf. Interval (95%)	
Type	−2.31	0.43	−5.32	0.00	−3.18	−1.44
Constant	6.43	0.31	20.74	0.00	50.81	7.05

*F(1, 59) = 28.32; *p* = 0.00, Adjusted R^2^ = 0.21

After regressing the screw displacement with respect to miniscrew type, force angulation and MIT, a statistically significant effect of force angulation (0.06 ± 0.02) and MIT (−0.01 ± 0.02) regressors was detected (Table 4). No effects of miniscrew type were observed (0.02 ± 0.02, *p* = 0.236).

**Table 4:** Linear regression model for miniscrew’s displacement (mm) at a force of 20 N by maximum insertion torque (MIT) and angulation of applied force

Miniscrew Displacement Linear Model**						
Covariates	$\widehat{\beta}$_i_	$\widehat{\sigma}$_i_	t	*p*_i_	Conf. Interval (95%)	
Angulation	0.06	0.02	3.33	0.00	0.02	0.09
MIT	-0.01	0.00	−3.14	0.00	−0.02	0.00
Constant	0.39	0.02	16.60	0.00	0.35	0.44

**F(2, 58) = 9.17; *p* = 0.00, Adjusted R^2^ = 0.21

## Discussion

Primary stability is defined as the mechanical stability in the bone immediately after miniscrew insertion. It is a function of the screw diameter and length, the number and design of the threads, the cortical thickness and the cortical bone density. It can be subjectively assessed by a clinician or evaluated by means of different quantitative methods (periotest, resonance frequency analysis, pullout test or insertion/removal torque recording) [7]. Many authors used insertion torque as a method to evaluate mechanical stability of miniscrews in an indirect way [1, 11–13], either with mechanical torque gauges and digital torque sensors.

In the present study, a digital torque sensor was used, as this type of instruments offers greater precision compared to other clinical devices, which are subject to errors related to axial loads, position and posture of the clinician and can be damaged over time [14]. Also, a displacing force was applied either at 90° or 45° to directly measure the dislocation of the miniscrews and then evaluate their stability under loading conditions. It was decided to apply a dislocating force with such angulations, instead of a pull-out test with the force applied along the miniscrew’s long axis, to mimic the orthodontic forces that are used in a clinical environment, which are usually applied perpendicular to the miniscrew’s long axis at various angulations. The amount of elastic deformation of the orthodontic wire had been pre-determined and eliminated. Even if the wire ligatures were tied manually, they were made by the same calibrated operator thus reducing variability in wire tightening. In addition, to evaluate the behavior of miniscrew under orthodontic loading, the presence of an elastic tie should be considered, since it is the way they are employed clinically. Concerning this matter, it is worth underlining that the purpose of the present study was not to assess absolute values of displacement, but to analyze comparatively the different behavior of two different miniscrew types under the same experimental conditions.

According to Motoyoshi et al. [8], an ideal insertion torque should be in a range between 5 and 10 N·cm in order to achieve higher success rate. However, a systematic review [7] revealed that no evidence exists to support this statement, and to recommend any value of insertion torque as more efficient. Heidemann et al. [15] found that, when pre-drilling of the implant site is performed, the pilot hole should not have a diameter greater than 80% of that of the screw in order to maintain good primary stability and an ideal insertion torque. In this study, the mean MIT value for self-tapping screws (4.12 ± 1.21 N·cm) was smaller than the mean MIT value for self-drilling screws (6.43 ± 2.09 N·cm); with regards to self-tapping miniscrews the diameter of the pilot drill was equal to 75% of the mini-implant’s one.

The two used miniscrew types differed —except for the shape of the tip— only for the outer diameter (2 mm for self-tapping miniscrews, 1.75 mm for self-drilling ones, whilst both miniscrew types had the same inner diameter of 1.3 mm, therefore the self-tapping miniscrews had greater threads’ depth). The different outer diameter is a consequence of different design requirements (self-drilling miniscrews bore themselves into the bone without any pre-drilling, whilst self-tapping ones advance into a pre-drilled pilot hole). Even if in case of comparison among miniscrews of the same design this could be considered a confounding factor [16], the present study was aimed to compare the behavior of self-drilling and self-tapping miniscrews, taking into account the differences related to the design of each device.

Regarding the definition of bone-like supports, several considerations were taken into account: human cadaver bone or fresh animal bone suffer quality modifications over time, and selecting various bone blocks of the same quality represents a highly variable operation. To avoid any possible bias due to differences in bone quality, synthetic bony blocks made of rigid polyurethane foam were used instead of fresh bone [2, 17–19]. According to international standards [20] the uniformity and consistent properties of rigid polyurethane foam make it an ideal material for comparative testing of bone screws and other medical devices and instruments.

Although orthodontic forces usually range between 0.3 and 4.0 N [1, 21], the value of 20 N was chosen to be representative of the general behaviour of the screw under loading conditions: in fact the goal of the present study was to evaluate differences between self-tapping and self-drilling miniscrews, not to define absolute properties. The orthodontic forces clinically used to load miniscrews are not sufficient to provoke their immediate displacement, while forces measured during pullout tests are usually above 100 N and don’t have a clinical meaning, but are used to describe absolute mechanical properties [17–19].

According to our results, there is a strong correlation between MIT and miniscrew type, probably due to the mini-implant design [17] as well as to the insertion technique, since the use of a pilot drill reduces the insertion torque [15]. Interestingly, while according to literature the greater outer diameter and deeper threads should provide greater MIT and stability [17, 22, 23], in the present experimental setup the self-tapping screws that had an outer diameter of 2.0 mm and an inner diameter of 1.3 mm showed lower MIT values compared to the self-drilling screws having an outer diameter of 1.75 mm and an inner diameter of 1.3 mm. Therefore, the main reason for the MIT values measured can be probably ascribed to the insertion technique.

When evaluating the stability of the screw by applying a displacing force, self-drilling screws showed a better behaviour (0.30 ± 0.04 mm at an angle of 90°) than self-tapping ones (0.35 ± 0.09 mm at an angle of 90°), and this difference was statistically significant (*p* < 0.05). Both miniscrew types showed better performances at an angle of 90° than at 45° in the opposite direction of the applied force, and this result was similar to those obtained by other authors [2]. Although statistically significant, the differences between the two groups were relatively small. Therefore, further clinical studies are needed to evaluate the impact of such different performances in a clinical environment.

On the basis of these results self-drilling screws look as preferable since they can be placed with higher insertion torque and therefore have greater primary stability. However, while this is true for this in vitro experimental set, in a clinical situation more variables have to be taken into account when pursuing an optimal primary stability and clinical success. The quality of the cortical bone is critical for miniscrew success [24, 25], but when inserting a screw in a thick or dense cortical layer micro-cracks or heat-damage can occur and cause bone resorption, which leads to a failure of the screw [26–29]: in these cases, for example, pre-drilling of the insertion site may be useful. In two clinical studies comparing self-drilling and self-tapping orthodontic miniscrews [10, 30], no statistically significant difference was found between them in terms of success/failure rate.

However, the use of a drill increases the risk of root or nerve damage, and self-drilling screws are simpler to manage (decrease in operative time, little bone debris, lower morbidity, and minimal patient discomfort as pre-drilling is not required).

According to the results of this study, it is possible to say that the tested self-drilling screws can achieve higher primary stability. However, self-drilling screws cannot be considered as absolutely preferable: a higher MIT is not always desirable, and the choice of the device depends on bone quality and quantity and the specific clinical situation.

## Conclusions

Force angulation and MIT have a statistically significant effect on miniscrews stability. An angle of 90° between miniscrew and loading force is preferable. Under the same conditions of bone-like support and same inner diameter, the tested self-drilling orthodontic miniscrews showed higher MIT and greater resistance against dislocation than the self-tapping ones.

## Additional file

Additional file 1: Dataset reporting the measurements of MIT and displacement for all the miniscrew samples. (XLSX 9 kb)

## Abbreviations

MIT: maximum insertion torque
TAD: temporary anchorage device
